# *GNAO1* encephalopathy: further delineation of a severe neurodevelopmental syndrome affecting females

**DOI:** 10.1186/s13023-016-0416-0

**Published:** 2016-04-12

**Authors:** Anna Marcé-Grau, James Dalton, Javier López-Pisón, María Concepción García-Jiménez, Lorena Monge-Galindo, Ester Cuenca-León, Jesús Giraldo, Alfons Macaya

**Affiliations:** Grup de Recerca en Neurologia Pediàtrica, Vall d’Hebron Institut de Recerca, Universitat Autònoma de Barcelona, Pg Vall d’Hebron 119-129, 08035 Barcelona, Spain; Laboratory of Molecular Neuropharmacology and Bioinformatics, Institut de Neurociències and Unitat de Bioestadística, Universitat Autònoma de Barcelona, 08193 Bellaterra Barcelona, Spain; Sección de Neuropediatría, Hospital Universitario Miguel Servet, P° Isabel la Católica 1,3, 50009 Zaragoza, Spain; Sección de Metabolismo, Hospital Universitario Miguel Servet, P° Isabel la Católica 1,3, 50009 Zaragoza, Spain; Secciones de Neuropediatría y Metabolismo, Hospital Universitario Miguel Servet, P° Isabel la Católica 1,3, 50009 Zaragoza, Spain; Pediatric Neurology Section, Hospital Universitari Vall d’Hebron, Pg Vall d’Hebron 119-129, 08035 Barcelona, Spain

**Keywords:** Early infantile epileptic encephalopathy, *GNAO1*, Exome sequencing, Ketogenic diet

## Abstract

**Background:**

*De novo* heterozygous mutations in the *GNAO1* gene, encoding the Gα o subunit of G-proteins, are the cause of a severe neurodevelopmental disorder, featuring early infantile seizures, profound cognitive dysfunction and, occasionally, movement disorder (early infantile epileptic encephalopathy-17).

**Methods:**

We report a further case of this association in a 20 month-old Spanish girl with neonatal-onset refractory seizures, progressive microcephaly, oral-lingual dyskinesia and nearly absent psychomotor development. We performed whole-exome sequencing, a computational structural analysis of the novel gene variant identified and reviewed the previously reported cases.

**Results:**

Trio whole-exome-sequencing uncovered a *de novo* p.Leu199Pro *GNAO1* mutation. Computational structural analysis indicates this novel variant adversely affects the stability of the G-protein heterotrimeric complex as a whole. Of note, our patient showed a sustained seizure reduction while on a ketogenic diet.

**Conclusions:**

With this observation, a total of twelve patients with *GNAO1* encephalopathy have been reported. Oral-lingual dyskinesia and responsiveness of seizures to ketogenic diet are novel features. The distorted sex ratio (12/12 females) of the condition remains unexplained; a differential gender effect of the disruption of G-protein- mediated signal transduction on the developing brain can be hypothesized.

**Electronic supplementary material:**

The online version of this article (doi:10.1186/s13023-016-0416-0) contains supplementary material, which is available to authorized users.

## Background

Epileptic encephalopathy is a group of neurological disorders characterized by severe and progressive cognitive and behavioral impairments, which are most probably caused or worsened by epileptic activity [1]. Heterozygous mutations in the *GNAO1* gene (MIM*139311), encoding the alpha subunit of the heterotrimeric guanine nucleotide-binding proteins (G proteins), were first described as a cause of early infantile epileptic encephalopathy (EIEE) in 2013 [2]. Subsequent reports have broadened the spectrum of clinical presentation [3–5] which includes prominent dyskinesia and intellectual disability with few or no seizures (4) and the condition is currently classified as EIEE17 (MIM #615473). Interestingly, all the cases reported to date involve female subjects despite the disease-causing gene maps to chromosome 16q13.

The *GNAO1* gene product is the alpha subunit of Go, a member of the G-protein family, involved in cellular signal transduction. Three functional G-protein subtypes are defined, namely inhibitory G-proteins (Gi), stimulatory G-proteins (Gs) and “other” (Go) the latter being abundantly expressed in the brain [6]. Typically, G-proteins are characterized by their alpha subunit, which binds and hydrolyzes GTP and interacts with specific GPCRs (G-protein coupled receptors) or effector molecules, such as adenylate cyclase. Relevant to neuronal excitability, Go proteins modulate neurotransmitter release by mediating the presynaptic auto-inhibitory effect of several neurotransmitters on their receptors, including M2/M4 muscarinic, alpha(2) adrenergic, mu/delta opioid and GABA-B receptors [7].

We here report the first Spanish girl with this disorder carrying a de novo *GNAO1* heterozygous mutation. State-of-the-art computational analyses suggest that this novel variant causes significant defects in protein structural stability.

## Methods

### Clinical report

The patient is a 21 month-old girl with a severe encephalopathy presenting with neonatal seizures. She is the second child of unrelated, healthy parents. A 4 year-old sister is healthy. Pregnancy and delivery were uneventful. Neonatal physical examination was reportedly normal, with head circumference of 34 cm (35th percentile). Seizures appeared on the third day of life and were tonic generalized or manifested as facial congestion, drooling, or motor phenomena such as sucking, grimacing or winking. They lasted from seconds to a few minutes, recurred up to 40 times per day and were refractory to treatment, including a pyridoxal phosphate-containing vitamin cocktail.

**Fig. 1:**
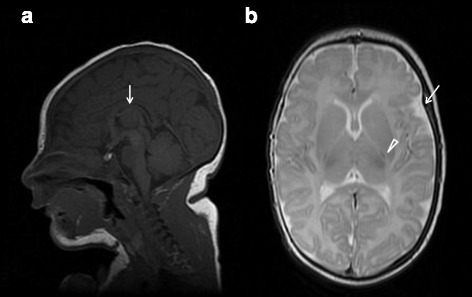
Brain MRI at age 18 days. Sagittal T1WI view showing thinning of the corpus callosum (arrow), with anterior predominance, and some degree of frontal lobe volume reduction (**a**). Axial T2WI view showing asymmetric enlargement of temporal subarachnoidal spaces (arrow) and mildly prominent sulci over the cerebral convexity, suggesting incipient atrophy. Myelination is delayed with minimal signals observed at the posterior limb of the internal capsule (arrowhead) and corpus callosum (**b**)

She was referred for study at the age of 3 months. The physical examination revealed a head growth deceleration (head circumference at 38,8 cm, 3rd percentile), low-set ears without other dysmorphic features, drowsiness with lack of visual fixation when awake, decreased muscle tone, reduced spontaneous movements and occasional startle. Interictal EEG recordings showed diffuse background slowing, and multifocal high-voltaged sharp waves and spike and slow-wave complexes, with predominance over both central-parietal areas. An extensive metabolic workup, including CSF lactate, amino acids and neurotransmitter metabolites, array CGH, fundoscopic examination and visual evoked potentials were normal. A previous brain MRI study, performed at 18 days of life, showed signs consistent with mild cerebral atrophy, thin corpus callosum and delayed myelination (Fig. 1).

On follow-up, EEG’s continued to show a slowed and high-voltaged background activity with poor differentiation and absent sleep elements plus very abundant, almost constant, bilateral sharp wave elements, consistent with severe epileptic encephalopathy. Seizures persisted unchanged despite the use of multiple antiepileptic drugs, including phenobarbital, valproate, levetiracetam, vigabatrin, carbamazepine, zonisamide, clobazam, topiramate and lacosamide in different combinations as well as a short trial with hydroaltesone. Although a slight improvement in seizure frequency was eventually obtained with a carbamazepine, topiramate and clobazam at age 11 months she was still suffering 15–20 seizures per day, mostly tonic generalized with upgaze deviation. With the institution of a 1:3 ketogenic diet (KD) seizure frequency progressively diminished and she remained seizure-free for almost two months. However, non-compliance with the KD resulted in recurrence of seizures. She currently averages 1–4 seizures per day. At age 19 months an EEG performed during sleep captured two seizures with, respectively, right and left mesial frontal origin. At age 21 months, the patient is severely disabled; she has not attained cephalic control or visual fixation and does not respond to any social cues. On examination, head circumference is 43 cm (<−3SD), height 86 cm (33th percentile) and weight 14 Kg (96th percentile). There is severe axial hypotonia, spastic tetraparesis, brisk deep tendon reflexes and paucity of voluntary movements. She has recently begun to display dyskinetic buccolingual movements, but without evidence of adventitious movements involving the trunk or limbs.

### Genetic studies and whole-exome sequencing

Peripheral blood samples were collected from the trio (patient and both progenitors). Genomic DNA was isolated using a standard salting-out method. The study was approved by the Vall d'Hebron University Hospital Institutional Review Board and informed consent was obtained from the patient’s parents according to the Helsinki declaration.

Exome sequencing of DNA samples from the trio was performed at the Centre Nacional d’Anàlisi Genòmica (CNAG) in Barcelona. 1 μg of genomic DNA was fragmented with an ultra sound device (Covaris, Woburn, MA, USA), denatured and hybridized with capture oligos (NimbleGen SeqCap EZ Exome v3.0 exome enrichment kit, Roche, Madison, WI, USA) which enriches for ~44 Mb of the human exonic regions. Captured sequences were enriched with streptavidin-conjugated paramagnetic beads. End repair, A-tailing, Illumina adapter ligation and post-capture amplification were done with TruSeq PE Cluster Kit v3, Illumina Corp, La Jolla, CA). Each captured exome was sequenced in one HiSeq2000 lane using version 3 chemistry. Coverage over 30X -was achieved for more than 80 % of the target. Alignment and variant calling were carried out following the GATK best practices for whole-exome sequencing. Sequencing reads were aligned to b37-decoy reference using BWA (http://bio-bwa.sourceforge.net/), then we marked duplicates, performed local realignment around indels, recalibrated the base quality scores, called variants with HaplotypeCaller and filtered them using Variant Quality Score Recalibration (VQSR) and hard filters. Refinement was done considering the pedigree, the population frequencies from Exome aggregation consortium (http://exac.broadinstitute.org/) [8], NHLBI Exome Sequencig Project (http://evs.gs.washington.edu/EVS/) [9], 1000 genomes project (http://www.1000genomes.org/) [10] and dbsnp (http://www.ncbi.nlm.nih.gov/SNP/) [11] and finally, the functional annotation (SnpEff, SnpSift) and the disease. In a first tier analysis (diagnostic exome sequencing) we investigated any variants within genes previously associated with epilepsy. This was followed by consideration of variants in any other gene covered by WES (research exome sequencing). Both, *de novo* mutations and transmitted alleles were analyzed. All candidate selected *de novo* mutations were absent in ExAC, and passed visual inspection of alignment quality using the Integrative Genomics Viewer (IGV).

Sanger sequencing was performed to confirm the *GNAO1 de novo* missense variant identified through whole-exome sequencing. To that end, exon six from the proband and progenitors was amplified by Polymerase-Chain-Reaction (PCR), purified and sequenced using the BigDye Terminator cycle sequencing kit v3.1 and an automated sequencer ABI PRISM 3730 DNA Analyzer (Applied Biosystems, Foster City, CA, USA) (primer sequences and PCR conditions available upon request).

### Structural modeling and free-energy calculations

#### Homology modeling human Gαo protein

The human WT Gαo protein was homology modeled from the crystal structure of mouse Gαo (PDB id: 3C7K) with MODELLER [12] (residues 22–346, see Additional file 1: Table S1 for sequence alignment). The sequence identity between mouse and human protein is 98 %. During homology modeling, conserved residues were maintained in their original crystal structure rotameric orientation. The co-crystallized ligands GDP and MG were directly transferred from the crystal structure into the homology model. The WT homology model was energy-minimized in the AMBER14SB force-field [13] with 1000 steps of steepest descent and 100 steps of conjugate gradients in CHIMERA [14]. The p.Leu199Pro mutation was inserted into the WT homology model with CHIMERA by applying the most appropriate side-chain rotamer and then energy-minimizing the mutant protein as performed previously in the AMBER14SB force-field.

#### Calculation of free-energy change

The predicted free-energy change (ddG) of the p.Leu199Pro mutation in human Gαo was made using ROSETTA [15] with a conversion factor of 0.73 employed to convert Rosetta Energy Units to kcal/mol [16]. The program was executed in “high-resolution mode” with full protein flexibility in side-chains and backbone (without bound GDP) and with the following flags: “iterations” set to 50, “local_opt_only” set to false, “sc_min_only” set to false, “opt_radius” set to 12.0, and “fa_max_dis” set to 9.0.

#### Molecular dynamics simulations

Both Gαo WT and Gαo Leu199Pro molecular dynamics (MD) systems were built using Amber LEap from AmberTools14 [13]. Ions were added to neutralize both systems and salt was added at a concentration of 0.2 M in each. Each system was then equilibrated with MD using ACEMD (Accelerating Biomolecular Dynamics in the Microsecond Time Scale) [17] at 300° and a pressure of 1 atm (NPT) in the AMBER14SB force-field for a total of 7.0 ns with harmonic restraints on the protein progressively relaxed from 10 kcal mol^−1^ Å^−2^ to 0 kcal mol^−1^ Å^−2^. Production run MD simulations of 1000 ns were then performed for both WT and mutant Leu199Pro proteins at 300° (NVT) in the AMBER14SB force-field using ACEMD [17]. VMD 1.9.2 [18] was used for RMSD (root-mean-square deviation) analyses of MD simulations, including heat-map generation.

## Results

### Whole-exome sequencing

**Fig. 2:**
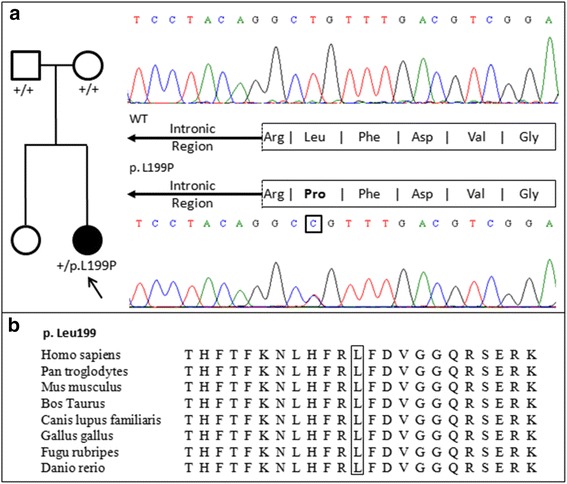
**a** Left, pedigree of the affected patient, showing the carrier of the heterozygous of the *de novo* mutation (filled symbol) and her unaffected sister and parents. Right, validation of the identified mutation by means of PCR and Sanger sequencing: electropherograms show the wild-type sequence in the progenitors (top) and the novel *GNAO1* c.596 T > C (NM_020988.2) variant in the proband (bottom), producing the amino acid change p.Leu199Pro (NP_066268.1). **b** Multiple sequence alignment of the GNAO1 protein region containing Leucine 199 (NP_066268.1) illustrating the high degree of evolutionary conservation of the affected residue

Whole exome sequencing from the patient and her parents (trio-based variant analysis) identified a single *de novo* variant within the epilepsy candidate genes, a heterozygous mutation GRCh37/hg19:chr16:56370645, NM_020988.2: c.596 T > C in *GNAO1*, producing the amino acid change p.Leu199Pro (NP_066268.1) in the proband. Read depth at the variant position was 38X, 42X and 50X for the proband and progenitors, respectively. The mutation was confirmed by PCR and Sanger sequencing (Fig. 2). This variant has not been previously described as related to the phenotype and is not present in any of the public genomic databases listed above. *In silico* prediction sofwares (SIFT [19], Mutation Taster [20] andPolyPhen-2 [21]) classified the novel variant as damaging. Despite the location of the variant in close proximity to a splice junction, the ESEfinder [22, 23] and Netgene2 [24, 25] scores did not indicate an effect on splicing (data not shown).

### Computational structural analysis

**Fig. 3:**
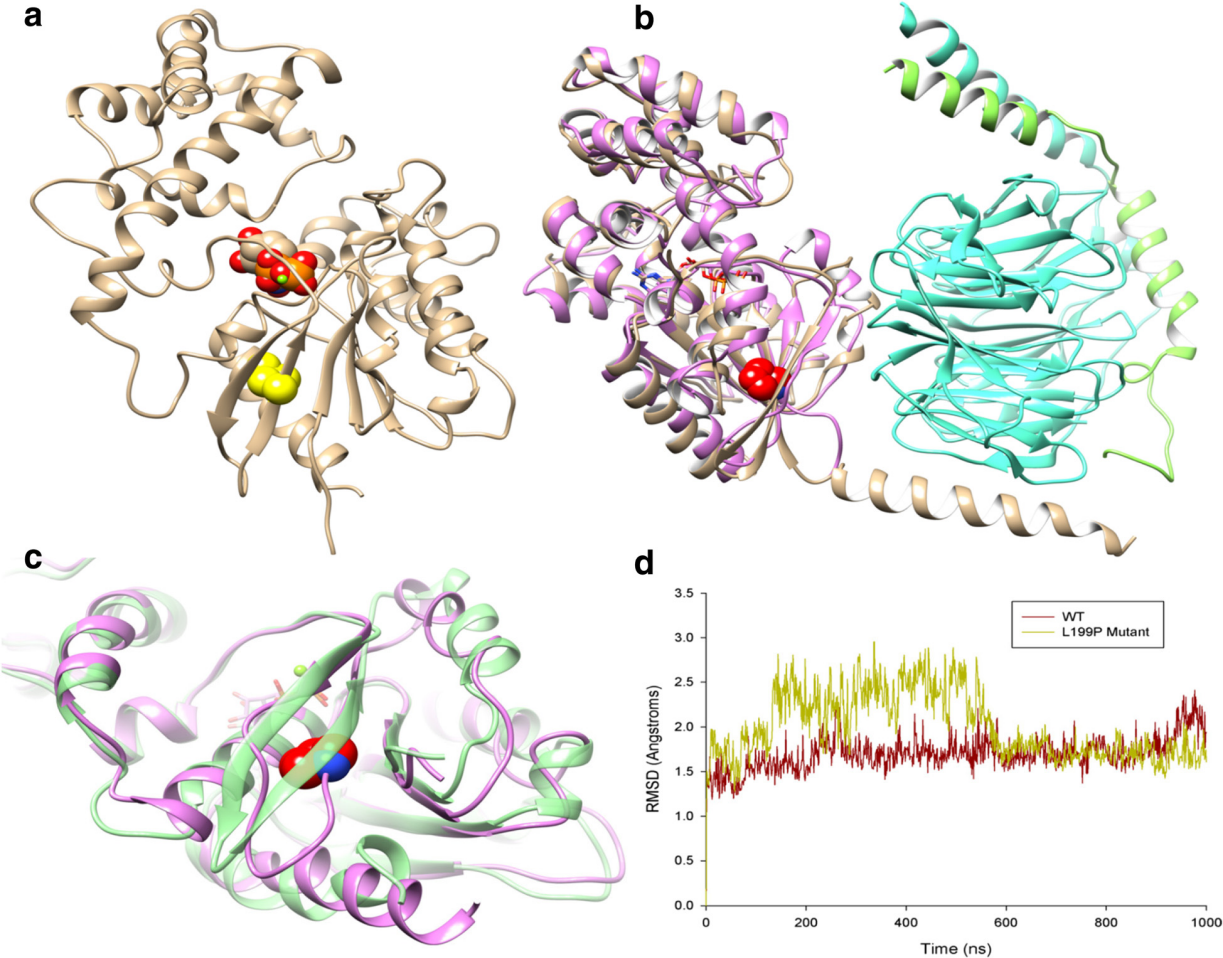
**a** Homology model of human WT Gαo. The mutated Leu199 is highlighted in yellow space-fill, protein backbone with secondary structure in beige, GDP in red/orange space-fill, magnesium in green. **b** Structural superposition of mutant Leu199Pro Gαo protein after 1 μ-second of MD simulation (in pink with mutation Leu199Pro shown as red space-fill) and heterotrimeric guanine-nucleotide-binding protein Gi (PDB id: 1GG2, Giα in beige, Giβ in cyan, Giγ in green). **c** GTPase domain of mutant Leu199Pro Gαo (in pink) compared to WT (in green) after 1 μ-second MD simulations (mutation Leu199Pro shown in red space-fill). **d** Plot of RMSD (**a**) for Cα atoms of WT Gαo protein and Leu199pro mutant during 1 μ-second MD simulations (RMSD comparisons made against initial structure)

The human WT Gαo protein with bound GDP was homology modeled from the crystal structure of mouse Gαo (PDB id: 3C7K) using MODELLER [8]. As these proteins share 98 % sequence identity, homology modeling accuracy can be considered very high (see Methods for details and Supplementary Information). The Leu199Pro mutation was introduced with CHIMERA [14]. The Leu199Pro mutation site is located in the GTPase domain of Gαo, specifically residing in the interior of a β-sheet that is packed between the N- and C-termini and interacting with adjacent α-helices. According to the homology model of human Gαo, Leu199 is not involved in GDP/GTP binding so its mutation to proline is not predicted to directly affect ligand binding or hydrolysis (see Fig. 3a).

The WT and mutant forms of human Gαo were analysed with ROSETTA [15] to calculate the predicted free-energy change (ddG) of the mutation. Using a conversion factor of 0.73 to convert from Rosetta Energy Units to kcal/mol [12], the predicted ddG of Leu199Pro is +6.1 kcal/mol. This suggests the mutation has a destabilizing effect on the protein and could possibly cause defects in its folding. To investigate this effect in more detail, both the WT protein and Leu199Pro mutant were subjected to a 1 μ-second MD simulation using ACEMD [13]. During these simulations, the WT protein remains stable in terms of both its secondary and tertiary structure with an RMSD that fluctuates between 1.0 and 2.0 Å (Fig. 3d). However the Leu199Pro mutant displays significant structural change in its GTPase domain, with multiple H-bonding in its central B-sheet broken, causing the creation of a large flexible loop region and increased positional fluctuation (Fig. 3c), with a protein RMSD of 1.5–3.0 Å across the simulation (Fig. 3d). In addition, the mutation also affects other areas of the domain, in particular α-helices: 208–217 and 257–262, both which lose their structural integrity and experience greater positional fluctuation during the simulation (Fig. 3c).

**Fig. 4:**
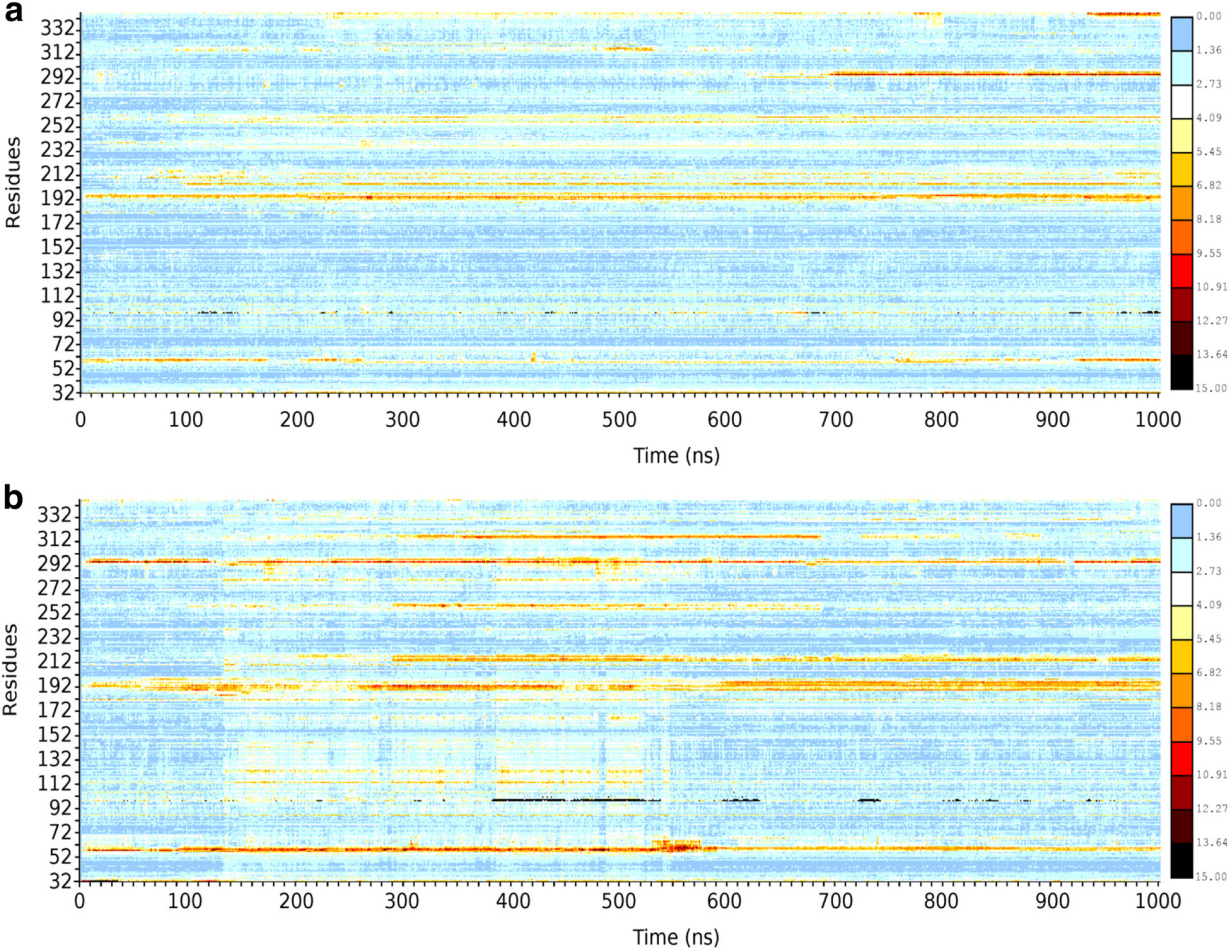
**a** RMSD per residue heat-map (scale 0–15, from low/blue to high/red) for WT Gαo throughout a 1 μ-second MD simulation. **b** RMSD per residue heat-map for mutant Leu199Pro Gαo. RMSD comparisons made against respective initial structures

In the context of the protein as a whole, heat-map representations of positional fluctuation reveal structural disturbances across both domains, GTPase and helical, in the mutant Leu199Pro protein (see Fig. 4). These disturbances occur in areas already described in the GTPase domain but also in several loop regions throughout the protein.

## Discussion

Early infantile epileptic encephalopathy-17 is a severe neurological disorder characterized by onset of intractable seizures in the first weeks or months of life, usually associated with EEG abnormalities, caused by heterozygous mutations in *GNAO1*. Neurological outcome is uniformly poor in all the infants described thus far. Imaging may disclose indistinct brain abnormalities, such as cerebral atrophy, delayed myelination, and/or or thin corpus callosum.

Including the present report, twelve patients with *GNAO1* encephalopathy have been described (Table 1). Early-onset epileptic encephalopathy has been the most frequent presentation (*n* = 9), but two out of the eight described Japanese patients, showed either few, late-onset seizures or none at all [4]. Clinical-EEG patterns are variable, and patients are referred to as suffering from Ohtahara, malignant migrating partial epilepsy of infancy, West syndrome or other forms of early epileptic encephalopathy phenotype [2–5]. Some patients show involuntary movements, including chorea, dystonia, athetosis or stereotypies [2, 4].

**Table 1:** Clinical features of 12 reported patients with GNAO1 encephalopathy

Case (ref)	Sex	Age	Age at seizure onset	Seizures type at onset	Epileptic syndrome	Dyskinesia	MRI findings	Global outcome	Epilepsy outcome	EEG features	Mutation	Inheritance
1 (2)	F	13y	4d	Tonic	OS	-	Normal (1mo), atrophy (5y)	Profound GDD	Intractable seizures	BS, multifocal	c.836 T > A (p.Ile279Asn)	*de novo*
2 (2)	F	4y	29d	Tonic	OS	-	DM, thin corpus callosum (10mo)	Profound GDD	Intractable seizures	BS, HS	c.521A > G (p.Asp174Gly)	*de novo* ^*a*^
3 (2)	F	Died 11mo	2w	Tonic/spasms	OS	-	Normal (3 mo)	Profound GDD	Intractable seizures	BS, HS	c.572_592del (p.Thr191_Phe197del)	*de novo*
4 (2)	F	8y	7mo	Opisthotonic	EE	+	DM (1y), thinning white matter, corpus callosum (4y)	Profound GDD	Intractable seizures	Diffuse SSW discharges	c.607G > A (p.Gly203Arg)	*de novo*
5 (3)	F	3y	3mo	Infantile spasms	EE	-	Mild atrophy	Severe to profound GDD	Seizure-free since age 5 m	HS	c.808A > C (p.Asn270His)	*de novo*
6 (3)	F	9y	3d	Tonic	EE-neonatal	-	DM, thinning of white matter	Severe to profound GDD	Daily tonic seizures	BS, HS	c.824 T > C (p.Phe275Ser)	*de novo*
7 (4)	F	20mo	2mo	Infantile spasms	EE	+	Cerebral atrophy, thin corpus callosum (10mo)	Profound GDD	Intractable CPS	HS, multifocal	c.680C > T (p.Ala227Val)	*de novo*
8 (4)	F	14mo	7d	Tonic-clonic	EE-neonatal	+	Normal (20d), cerebral atrophy, DM (14mo)	Profound GDD	Intractable CPS	SW bursts, migrating focal, multifocal discharges	c.607G > A (p.Gly203Arg)	*de novo*
9 (4)	F	13y		No seizures	-	+	Normal (4y, 12y)	Profound GDD		Normal	c.736G > A (p.Glu246Lys)	*de novo*
10 (4)	F	18y	10y	Complex partial	-	+	Global atrophy, thin corpus callosum (11y, 14y)	Severe GDD	Complex partial seizures (11y)	Normal at 4 years, later diffused low	c.625C > T (p.Arg209Cys)	*de novo*
11 (5)	F	4y	1mo	Myoclonic	OS	+	Asymmetrical subarachnoidal space in temporal regions, myelinization delay in corpus callosum	Profound GDD	Intractable seizures	BS, HS, multifocal	c.692A > G (p.Tyr231Cys)	*de novo*
Present report	F	20mo	3d	Tonic	EE-neonatal	+	DM, thin corpus callosum	Profound GDD	Seizure-free, ketogenic diet	Background slowing, multifocal	c.596 T > C (p.Leu199Pro)	*de novo*

*OS* Ohtahara syndrome, *EE* epileptic encephalopathy, *ID* intellectual disability, *BS* burst-suppression, *HS* hypsarrhythmia, *SSW* spike and slow wave, *DM* delayed myelination, *GDD* global developmental delay, *NA* not available, ^*a*^somatic mosaicism

Refractoriness to antiepileptic drugs appears a common feature in all patients displaying the EIEE phenotype. In our patient a complete cessation of seizures was noted one month after the institution of a ketogenic diet and the patient remains seizure-free five months into the diet. Considering the widely admitted deleterious effect of seizures on the developing brain, institution of this therapeutic modality early in the evolution of *GNAO1* encephalopathy seems advisable. Based on the loss of calcium-current inhibition found in vitro in *GNAO1* mutants, resulting from altered Gαo -mediated signaling induced by norepinephrine, Nakamura et al. suggested the use of selective calcium-channel blockers or the use of the high-voltage activated calcium channels modulator topiramate to alleviate epilepsy in patients with *GNAO1* mutations [2]. Our patient, at variance with previous cases where treatment was specified, did receive topiramate since the age of 8 months, but without noticeable improvement in seizures or changes in her developmental profile.

All twelve patients described carried heterozygous de novo missense mutations [2–5]. Indeed, the p.Leu199Pro is predicted to cause protein dysfunction in both of the described GNAO1 isoforms, according to the used in silico prediction tools. Furthermore, the application of the recently published ACMG guidelines for interpretation of sequence variants [26] to the p.Leu199Pro variant classified it as pathogenic by virtue of fulfilling one strong (PS2) plus two moderate (PM1, PM2) and two supporting (PP2,PP3) pathogenic criteria.

In vitro functional studies showed that three out of four analyzed missense *GNAO1* mutations resulted in abnormal, cytoplasmic localization of the protein, while electrophysiological studies proved that all four variants produced impaired N-type calcium channel current inhibition after norepinephrine application [2]. Interestingly, a mouse model harboring a heterozygous (p.Gly184Ser) *Gnao1* missense mutation displayed a severe and lethal epileptic phenotype that was absent in the *Gnao1* hemizygous knock-out mouse, suggesting that pathogenic *GNAO1* mutations may act through a gain-of-function, dominant effect [27].

In our present work, we investigated the effect of the novel p. Leu199Pro mutation in the structure of the human Gαo protein using state-of-the-art computational tools. These provide evidence that the mutation causes significant defects in protein stability. The observed disturbances occur locally in the GTPase domain where the mutation is situated but also in several loop regions throughout the protein. This suggests the p.Leu199Pro mutation decreases overall structural stability and perhaps folding of the Gαo protein. From a functional point of view, the location of the p.Leu199Pro mutation may be particularly significant in terms of interactions Gαo makes with its binding partner Gβϒ and others. With respect to the crystal structure of the complete heterotrimeric guanine-nucleotide-binding protein Gi (PDB id: 1GG2), when the Gαo subunit is superimposed, the mutation p.Leu199Pro is in direct contact with Gβϒ (see Fig. 1b). Therefore any introduced structural instability in this interface would likely adversely affect the stability of the Gαβϒ complex as a whole. From the observed results, it is certainly conceivable that the p.Leu199Pro mutation could generate this level of instability. The issue of a female gender bias in this disorder deserves special consideration. A distorted sex ratio can be found in many complex disorders, for instance autism spectrum disorder [28] and a male-biased effect has been described for 16p13.11 copy number variants as predisposing factor to a range of neurodevelopmental disorders [29]. Possible causes include hormonal factors, sexual dimorphic brain differences, imprinting or genetic modifiers, with a net effect of greater protection of the female brain. It is conceivable that the penetrance of *GNAO1* mutation is higher in the less resilient male brain thus producing a more severe phenotype. While female bias in EIEE-17 remains unexplained, it could be posited that disruption of G-protein signal transduction may lead to prenatal lethality in males.

## Conclusions

We describe a further female patient with *GNAO1*-associated neonatal-onset epilepsy and a severe neurodevelopmental disorder. Seizures were amenable to treatment with KD. Structural analysis of the novel mutation suggests it introduces significant structural instability into both the Gαo subunit and the whole G-protein heterotrimeric complex. The resulting defective cellular signal transduction is in all probability deleterious during brain development and putatively fatal in the male embryo.

## Additional file

Additional file 1: Table S1. Sequence alignment as calculated by NCBI-BLAST between human Gαo (Query) and mouse Gαo (Sbjct, PDB id: 3C7K). (DOC 69 kb)

## Abbreviations

ACEMD: accelerating biomolecular dynamics in the microsecond time scale
BWA: Burrows-wheeler aligner
CGH: comparative genomic hybridization
CSF: cerebrospinal fluid
ddG: predicted free-energy change
EEG: electroencephalography
EEIE: early infantile epileptic encephalopathy
GABA: gamma-aminobutyric acid
GATK: genome analysis toolkit
GDP: guanosine diphosphate
Gi: inhibitory G-proteins
Go: “other” G-proteins
GPCRs: G-protein coupled receptors
Gs: stimulatory G-proteins
GTP: guanosine triphosphate
IGV: integrative genome viewer
KD: ketogenic diet
MD: molecular dynamics
MG: magnesium
MRI: magnetic resonance imaging
PCR: polymerase-chain-reaction
PDB: protein data bank
RMSD: root-mean-square deviation
VQSR: variant quality score recalibration
WT: wildtype
