# Supplementary figures and images for: GNAO1 encephalopathy: further delineation of a severe neurodevelopmental syndrome affecting females
Source: Orphanet J Rare Dis. 2016 Apr 12;11:38. doi: 10.1186/s13023-016-0416-0 (PMC4830060; doi:10.1186/s13023-016-0416-0)

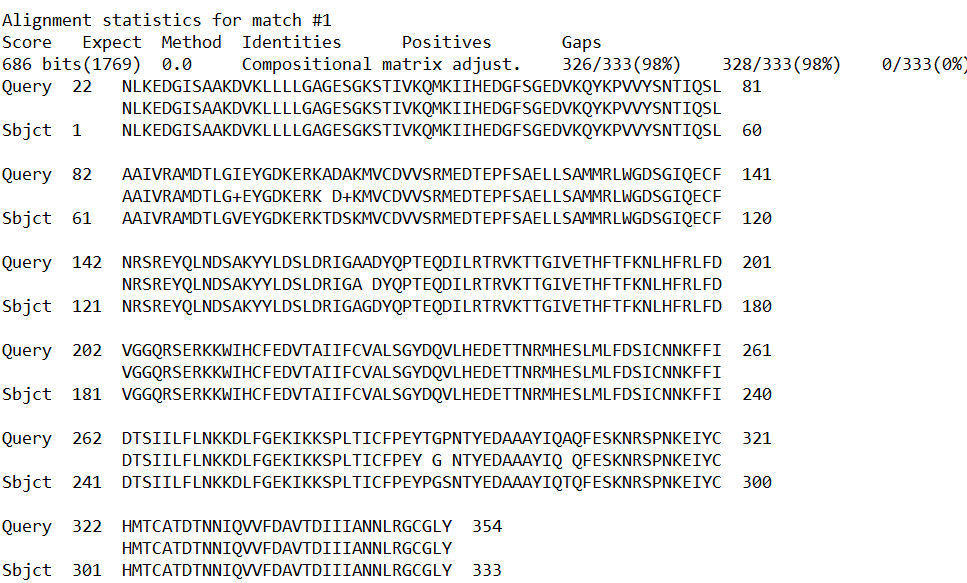

Supplement: Additional file 1: Table S1. — Sequence alignment as calculated by NCBI-BLAST between human Gαo (Query) and mouse Gαo (Sbjct, PDB id: 3C7K). (DOC 69 kb) [file 13023_2016_416_MOESM1_ESM.doc]
